# The benefits of haptic feedback in robot assisted surgery and their moderators: a meta-analysis

**DOI:** 10.1038/s41598-023-46641-8

**Published:** 2023-11-06

**Authors:** Max Bergholz, Manuel Ferle, Bernhard M. Weber

**Affiliations:** 1 Department of Ergonomics, Technical University of Munich, 85748 Garching, Germany; 2 Institute of Robotics and Mechatronics, German Aerospace Center, 82234 Wessling, Germany

**Keywords:** Health care, Medical research, Engineering, Mechanical engineering

## Abstract

Robot assisted surgery (RAS) provides medical practitioners with valuable tools, decreasing strain during surgery and leading to better patient outcomes. While the loss of haptic sensation is a commonly cited disadvantage of RAS, new systems aim to address this problem by providing artificial haptic feedback. N = 56 papers that compared robotic surgery systems with and without haptic feedback were analyzed to quantify the performance benefits of restoring the haptic modality. Additionally, this study identifies factors moderating the effect of restoring haptic sensation. Overall results showed haptic feedback was effective in reducing average forces (Hedges’ g = 0.83) and peak forces (Hedges’ g = 0.69) applied during surgery, as well as reducing the completion time (Hedges’ g = 0.83). Haptic feedback has also been found to lead to higher accuracy (Hedges’ g = 1.50) and success rates (Hedges’ g = 0.80) during surgical tasks. Effect sizes on several measures varied between tasks, the type of provided feedback, and the subjects’ levels of surgical expertise, with higher levels of expertise generally associated with smaller effect sizes. No significant differences were found between virtual fixtures and rendering contact forces. Implications for future research are discussed.

## Introduction

Robot assisted surgery (RAS) has shown the potential to lead to preferable clinical outcomes for patients^1^ while reducing the surgeon’s cognitive^2^ and physical workload^3^. Robot assistance has contributed to improving surgical interventions, especially in the field of minimally invasive surgery. Consequently, robotic surgery systems have seen broad adoption. For example, the majority of prostatectomies performed in the United States in 2010 utilized robotic assistance^4^.

An obvious drawback of RAS is the loss of haptic sensation, depriving surgeons of a natural source of information about interaction forces^5^. This shortcoming has been linked to diminished surgical performance^6^. Several current research and development programs try to rectify this drawback by artificially restoring haptic sensation to the surgeon using master–slave robotic systems^7–9^. The proposed solutions vary greatly in terms of the methods, how haptic feedback is provided to the surgeon, and their intended fields of application^10–12^. A general distinction can be made between direct haptic feedback and haptic sensory substitution. The latter provides information on forces via auditory^13^ or visual^14^ cues, whereas direct haptic feedback, which will be the subject of this study, aims to recreate haptic impressions as naturally as possible. The most common approach is kinesthetic haptic feedback, wherein the impedance incurred on the slave side of the robotic system is mirrored back to the master side. For example, Talasaz, Trejos, and Patel^15^ used a motorized master controller to mirror the impedance forces picked up by sensors in the robot’s end effectors during a robot assisted suturing task. This resulted in a reduction of the forces the subjects applied and thereby lowered risks of suture breakage. Alternatively, haptic feedback is provided via vibrations of the master device^16^ or via actuators stimulating the user’s fingertips^17^. This way, the illusion of holding an object in hand is created.

RAS is most studied in the context of laparoscopic surgery^18^, but other paradigms also increasingly employ robotic assistance. In retina surgery, for example, robotic assistance is utilized for the high accuracy and tremor-free stability a robotic end-effector provides. The feedback force can be upscaled to allow the surgeon to sense interaction forces below the human perception threshold^19^. Another motivation for using robots in the operating room is to shield the surgeon from harm. In venous catheterization procedures, for example, x-rays are commonly used for live imaging of the patient’s vascular system. Haptic feedback allows the surgeon to stay outside the operation room while retaining the haptic sensation crucial for preventing vascular puncturing^12^.

While the benefits of haptic feedback technology seem obvious, they must be set against the incurring costs, not only monetary but also in terms of the required additional space and maintenance procedures^20^. Some researchers also suggest that with adequate expertise, surgeons can extract sufficient information about interaction forces during surgery from visual feedback alone. The advanced stereoscopic view of modern robotic surgery systems might allow robotic specialists to gauge interaction forces from visible tissue deformation. It can even create a faux haptic sensation, potentially rendering true haptic feedback redundant^21^. Conversely, other researchers found benefits of haptic feedback even for very experienced surgeons^8^.

It is, therefore, pivotal to provide clinics with information about the expected benefits of haptic feedback systems to allow for informed decisions and to provide researchers with information on which approaches to haptic feedback are the most promising. Existing reviews are primarily qualitative in nature^22,23^, focused on a single system^24^ or no longer reflect the current state of development^25^. Given the field's broad and rapidly evolving nature, an expansive systematic analysis of recent research is needed. In this meta-analysis, key performance metrics were identified, and the overall effects of haptic feedback on these metrics and potential moderating factors were determined. Hence, the present study aims not just to quantify the general benefits of haptic feedback but also their extent under specific conditions, namely, given a particular surgical task, level of users’ expertise, and type of feedback. By identifying conditions under which haptic interaction technology is particularly useful, this study provides a basis for decision-making for practitioners and researchers.

## Methods

### Report compilation

This meta-analysis was performed following the guidelines laid out in the PRISMA statement^26^. Literature was compiled using several online data libraries. The databases PubMed, IEEEXplore, and Scopus were consulted. This selection has been chosen to cover both key disciplines relevant to the subject, namely medicine (PubMed) and engineering (IEEEXplore), as well as a more generalized database in the form of Scopus to additionally cover applicable papers outside these fields. Given the enormous diversity of paradigms and measures in the field, the search aimed to generate a large number of results rather than trying to minimize the number of irrelevant results. Hence, a broad search string was chosen to compile an as complete as possible body of studies.

$$ \left( {telerobotics \;OR \;robot \;assisted \;{\text{OR}}\;{\text{ telesurgery}}} \right){ }\;{\text{AND}}\; \left( {haptics{ }\;{\text{OR }}\;force\; feedback\;{\text{ OR}}\;{ }tactile} \right) $$

**Figure 1:**
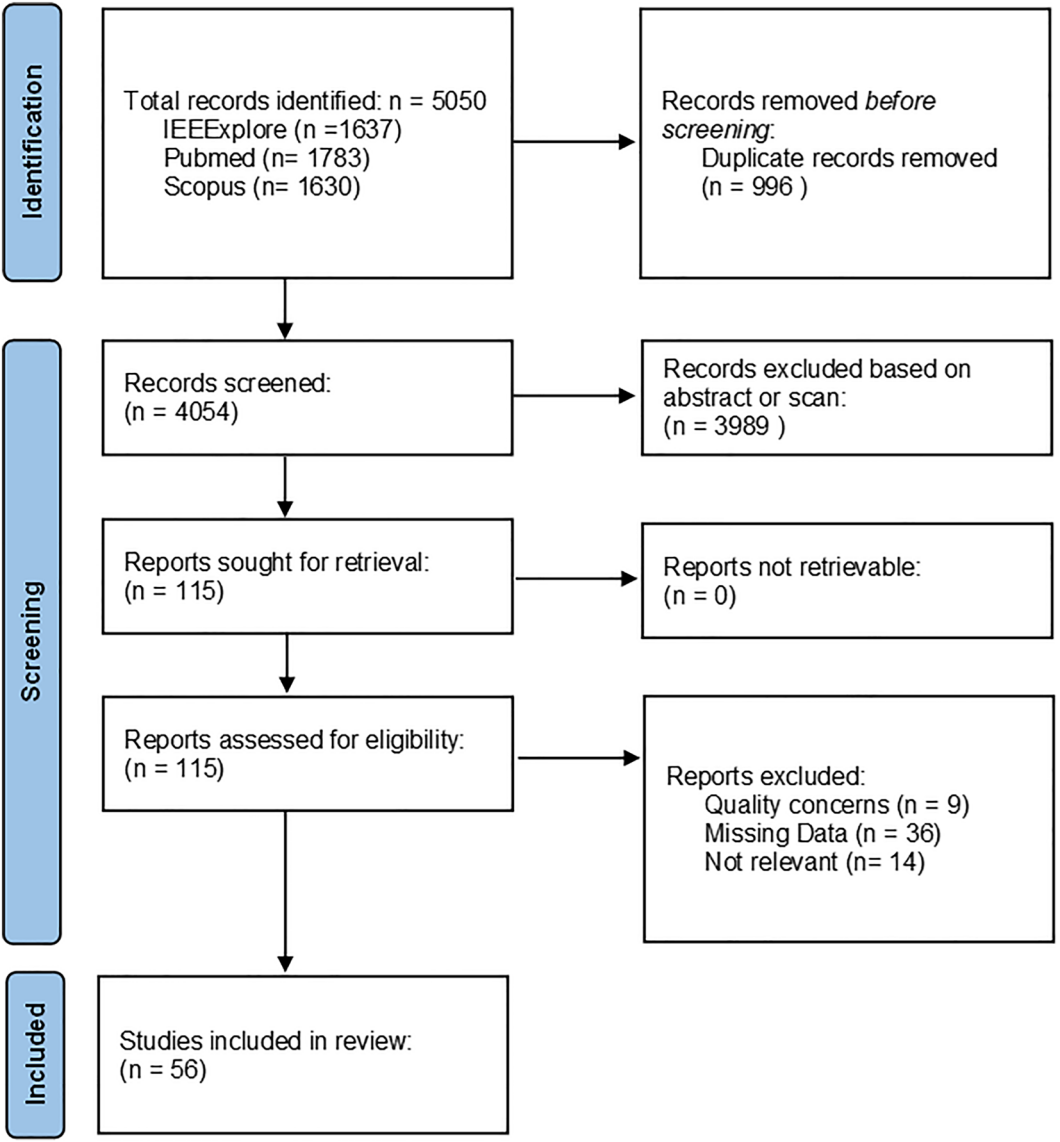
Flowchart visualizing the process of sample compilation.

The reports were compiled in September and October 2022. Papers published since 2013 were eligible, the last point in time covered in the most recent meta-analysis on haptic feedback in general RAS^25^. Additional criteria were defined to exclude non-peer-reviewed publications and to reduce the amount of overlap. This process yielded 1637 papers on PubMed, 1783 on IEEEXplore, and 1630 on Scopus, respectively. 996 of these 5050 results were removed as duplicates. The remaining 4054 papers were screened and filtered by the primary author (Fig. 1).

The following inclusion criteria were defined. The study needs to (1) be peer-reviewed, (2) report quantitative data, (3) feature a direct comparison of subjects’ performance with and without haptic feedback, (4) use a master–slave robot system with direct control, (5) be applicable in surgical contexts, (6) make use of direct haptic feedback, (7) be written in English or German. Notably, this excluded papers investigating sensory substitution^27^, haptic feedback in non-surgical contexts^28^, or the benefits of haptic feedback for training^29^. Filtering by these criteria eliminated most search results, leaving N = 115 papers, all of which could be retrieved in full and were consequently subjected to further scrutiny.

The following quality criteria were defined: (1) At least n = 2 subjects per study group, (2) The paper had to account for potential learning effects by counterbalancing or randomizing the order of trials, and (3) No authors participated as test subjects. Nine papers had to be excluded as they failed to meet these criteria. Additionally, 14 papers had been excluded because, upon closer inspection, their compliance with inclusion criteria became dubious. For example, technologically immature systems that would require substantial modification before surgical application were excluded^30^. 36 papers had to be excluded because of lacking data reporting. The final sample consisted of 56 primary studies with n = 768 individual subjects producing k = 174 observations. A complete list of included primary studies can be found in Table 1.

**Table 1:** Summary list of studies included in the meta-analysis.

First author	Year	Obser-vations	Measures	Subjects’ expertise	Task	Surgical field	Feedback type	VF /CF
Abiri^34^	2017	1	Time	Inexp	Grasping	Lapa	Cut	CF
Abiri^35^	2019a	4	Success, Time	Inexp	Palpation	Lapa	Kin, VT	CF
Abiri^36^	2019b	10	Avg F, Peak F	Exp, nov	Grasping	Lapa	Com, Cut, Kin	CF
Abiri^7^	2019c	1	Point Δ	Nov	Suture	Lapa	VT	CF
Aggravi^37^	2021	6	Success, point Δ, time	Inexp	Insertion	Lapa	Kin, VT	CF
Allebas^38^	2017	1	Avg F	Mix	Palpation	Lapa	Kin	CF
Bahar^39^	2020	6	Deg Δ, Peak F, time	n/a	Insertion	Lapa	Kin	CF, VF
Bao^40^	2018	1	Time	Exp	Catheter	Cardio	Kin	CF
Camara^41^	2018	1	Success	Inexp	Palpation	Onco	Kin	CF
Chauhan^42^	2017	1	Avg F	n/a	Grasping	Lapa	Kin	CF
Chinello^43^	2020	6	Avg F, point Δ, time	Inexp	Palpation	Lapa	Com, cut	CF
Chowriappa^44^	2013	2	Peak F, point Δ	n/a	Insertion	Lapa	Kin	CF
Currie^45^	2016	1	peak F	Mix	Suturing	Lapa	Kin	CF
Dagnino^46^	2018	3	Avg F, peak F, time	Inexp	Catheter	Cardio	Kin	CF
Dai^47^	2019	3	Avg F, point Δ. time	Nov	Suturing	Lapa	Kin	CF
Dalvand^48^	2014	1	Success	Inexp	Palpation	Lapa	Kin	CF
Diez^8^	2019	3	Avg F, peak F, time	Exp	Grasping	Lapa	Kin	CF
Ebrahimi^49^	2018	2	Avg F, time	Mixed	Tracing	Ophtha	Kin	CF
Ehrampoosh^10^	2013	3	Success	n/a	Palpation	Lapa	Kin	CF
Elayaperumal^10^	2014	1	Success	Inexp	Insertion	Lapa	Kin	CF
Fichera^50^	2015	2	Point Δ	Inexp	Laser	Tors	Com, Kin	VF
Francone^51^	2019	2	Avg F, time	Mixed	Grasping	Lapa	Kin	CF
Gambaro^52^	2014	2	Path Δ, time	n/a	Tracing	Lapa	VT	VF
Gerena^53^	2020	1	Success	n/a	Grasping	Lapa	Kin	VF
Gibo^54^	2014	1	Avg F	Inexp	Grasping	Lapa	Kin	CF
Gramma- tikopoulou^55^	2016	3	Path Δ, success	Novice	Grasping, scan	Lapa	Kin	VF
Howard^16^	2016	1	Point Δ	Novice	Suturing	Lapa	VT	CF
Jeong^56^	2021	6	Avg F, peak F, time	Inexp	Tracing	Ophtha	Kin	CF
Jin^57^	2021	2	Avg F, time	Inexp	Catheter	Cardio	Kin	CF
Jou^17^	2022	12	Avg F, Peak F	Exp, mix, nov	Section, grasping	Lapa	Cut	CF
Karponis^58^	2019	1	Point Δ	Nov	Insertion	Lapa	Kin	CF
Kim^11^	2016	1	Peak F	n/a	Grasping	Lapa	Kin	CF
Li^9^	2022	2	Avg F	Mixed	Catheter	Cardio	Kin	CF
Lim^59^	2015	3	Avg F	Inexp	Grasping	Lapa	Kin, cut	CF
Lopez^60^	2013	2	Path Δ, point Δ	n/a	Insertion	Lapa	Kin	VF
Mendelsohn^61^	2020	1	Time	Nov	Section	TORS	VT	CF
Molinero^62^	2019	1	Time	Exp	Catheter	Cardio	Kin	VF
Nakazawa^63^	2016	2	Point Δ, time	Inexp	Tracing	Neuro	Kin	VF
Navkar^64^	2013	1	Time	Inexp	Catheter	Cardio	Kin	VF
Olivieri^65^	2018	2	Path Δ, time	Exp	Laser	TORS	Kin	VF
Ouyang^66^	2021	3	Avg F, success, time	Mixed	Palpation	Lapa	Cut	CF
Pacchierotti^67^	2015	6	Avg F, time	Inexp	Grasping	Lapa	Com, Cut, Kin	CF
Pacchierotti^68^	2016	4	Deg Δ, time	Inexp	Palpation	Lapa	Cut, VT	CF
Portolés^69^	2015	2	Avg F, time	Inexp	Tracing	Lapa	Kin	CF
Power^70^	2015	2	Time	n/a	Grasping	Lapa	Kin	CF
Quek^71^	2019	6	Avg F, peak F	Inexp	Grasping	Lapa	Cut, Kin	
Saracino^72^	2019	24	Avg F, peak F, time	Exp, inexp, nov	Grasping, palpation, section	Lapa	Kin	CF
Saracino^73^	2020	3	Peak F, success, time	Nov	Palpation	Lapa	Kin	CF
Seung^74^	2017	3	Path Δ	Nov	Tracing, section	Neuro	Kin	VF
Song^75^	2017	2	Avg F, time	Inexp	Catheter	Cardio	Kin	CF
Tahir^76^	2022	2	Time	Inexp	Catheter	Cardio	Kin, VT	VF
Talasaz^15^	2017	4	Peak F, time	Mix	Insertion, suture	Lapa	Kin	CF
Urias^77^	2020	1	Avg F	Exp	Section	Ophtha	Kin	CF
Wottawa^32^	2016	4	Avg F	Exp, nov	Grasping	Lapa	Cut	CF
Yin^78^	2018	1	Time	Mix	Catheter	Cardio	Kin	CF
Zhang^12^	2021	2	Peak F, time	Mix	Catheter	Cardio	Kin	CF

“peak F”, peak forces; “avg F”, average forces. “Δ” , deviation; “exp” , experienced subj.; “nov” , novice subj.; “inexp”, inexperiecned subj. “mixed” , diffent levels of expertise in the same group; “n/a”, level of expertise not reported; “cardio” , cardiovascular; “lapa”, laparoscopy; “neuro” , neurosurgery; “onco”, oncology; “ophtha”, ophthalmology; ““TORS”, transoral robotic surgery. “com” , combined feedback; “cut” , cutaneous feedback; “kin” , kinesthetic feedback; “VT”, vibrotactile feedback. “VF”, Virtual Fixtures; “CF”, Contact Forces. Mixed and non-available levels of expertise and success rate outcomes were not considered for subgroup analysis.

### Data extraction

To calculate effect sizes, means and standard deviations had to be extracted. Where available, these metrics were taken directly from the publications. Otherwise, they were retrieved by the primary author by measuring graphics and figures provided in the primary studies using open-source image manipulation software (GIMP 2.10). This method ensured accuracy on the level of a single pixel and, thereby, the numerical value presented by a pixel. Where this was not possible either, effect sizes were estimated based on sample size and p-values^31^. When papers failed to report precise p-values, the authors of the primary studies were contacted with a request for the data measures in question. If the necessary data could not be retrieved this way either, the study had to be excluded.

### Statistical methods

All statistical analyses were performed in “Comprehensive Meta-Analysis Version 4” (CMA, Biostat Inc, Englewood, New Jersey, USA). The following performance metrics have been extracted from the primary studies: (1) Force applied (further divided into average force and peak force), with smaller forces being considered favorable as high forces are associated with increased tissue damage^32^. (2) Time required, with shorter times being considered desirable. (3) Accuracy, to gauge precision, several sub-measures were combined: deviation from a target angle, deviation from a target point, and deviation from a target path. Lower deviation is desirable; (4) Success rates; in the present data set, this measure chiefly refers to correct tissue identification in palpation tasks.

Since it can be expected that true effect sizes vary between primary studies, a random effects model was chosen. All measurements have been valence-coded such that higher values indicate more desirable outcomes (i.e., shorter times, lower applied forces, less deviation, and higher success rates). The cutoff for statistical significance was set to p = 0.05. Hedges’ g was chosen to calculate effect sizes rather than the more common Cohen’s d since the latter tends to overestimate the effect size, especially for small sample sizes^31^. Hedges’ g can thus be seen as more conservative as it adjusts Cohen’s d with the factor J.

$$ J = 1 - \frac{3}{4df - 1} $$

The size of effects can be interpreted analogously to Cohen’s d. That is, effect sizes larger than 0.8 are considered large. They are immediately evident to observers and practitioners and make a meaningful difference in clinical practice. On the other hand, effect sizes below 0.2 are considered irrelevant even if they are statistically significant^31^.

Heterogeneity was assessed with the Q-statistic, *I*^2^-statistic, and the prediction interval. Following recommendations by Medina et al.^33^ it is essential to differentiate these measures. The Q-statistic functions analogously to the F-statistic in primary studies in that it tests against the null hypothesis that all effects in the sample are of equal size. A significant Q-value indicates that not all observed variety between effect sizes results from random error, meaning the effect sizes are heterogeneous. Furthermore, the Q-statistic can be used to test whether the effect sizes observed in different subgroups significantly differ. *I*^2^ quantifies which percentage of the observed variety represents the heterogeneity of the true effect sizes. Lastly, the prediction interval (PI) gives the range of effect sizes 95% of individual effects in a comparable population are expected to fall into. It is similar to, but has to be differentiated from, the confidence interval (CI), which estimates the range the median effect falls into. The confidence interval is reported as well. In this study, the conventional 95% confidence interval was chosen.

### Subgroup analysis

Based on the reviewed literature, the following categorical variables have been determined as potential moderators and extracted: Level of subjects’ expertise, experimental task, type of haptic feedback, and the distinction between virtual fixtures and contact forces. These potential moderators will be briefly described in the following:

### Level of expertise

(1) “Inexperienced” denotes subjects with no prior surgical training. (2) “Novices” denotes subjects with medical training but no or limited, i.e., no more than a year of practical experience in the tested procedure. (3) “Experts” denotes subjects with extensive multiyear expertise on the specific procedure tested in the experiment.

### Task

Since surgeries often consist of various tasks that partly overlap between procedures, the investigated surgeries were broken down into more elemental tasks for this meta-analysis. The following tasks were extracted from the reviewed body of literature: (1) Catheterization: insertion of venous catheters into the vascular system; (2) Grasping: tasks consisting of grasping, holding, or pulling primarily of tissue, (3) Insertion: puncturing of tissue with needles and/or trocars, (4) Laser: cutting and/or sectioning of tissue involving surgical lasers, (5) Palpation: physical Investigation of tissue with the robot’s end effector, (6) Scan: Investigation of tissue via additional means such as ultrasound scans, (7) Section: cutting and/or carving of tissue, (8) Suturing: knot-tying and stitching of tissue, and (9) Tracing: following a line on the surface of a tissue sample.

### Feedback type

The different types of feedback employed by researchers to restore haptic sensation have been categorized as follows: (1) Kinesthetic feedback: application of a force vector to the master side of the robot mirroring the impedance offered by an object in contact with the slave side. (2) Vibrotactile feedback: provision of information to the user via the vibration of either a master controller or an externally worn device. (3) Cutaneous feedback: provision of haptics via the motion of actuators against the operator’s fingertips, creating an illusion of holding an object. (4) Combined: any combination of the above.

### Contact vs. virtual fixtures

(1) Contact forces: Feedback is given about contact with physical objects at the robotic site. (2) Virtual fixture: Feedback is given in response to virtual points, which have been defined pre-operatively in relation to either the patient’s body (e.g., a virtual point close to sensible tissue) or the posture of the robot (e.g., a certain degree on the slave-robot’s joints).

In addition to the moderators, we list the relevant surgical fields in the table below. While several studies are potentially applicable to multiple fields, an intended field of application can be noted for each paper. The following surgical fields were considered in the primary studies: (1) Cardiovascular surgery, (2) Laparoscopic surgery, (3) Neurosurgery, (4) Oncology, (5) Ophthalmology, and (6) Oncology.

## Results

**Figure 2:**
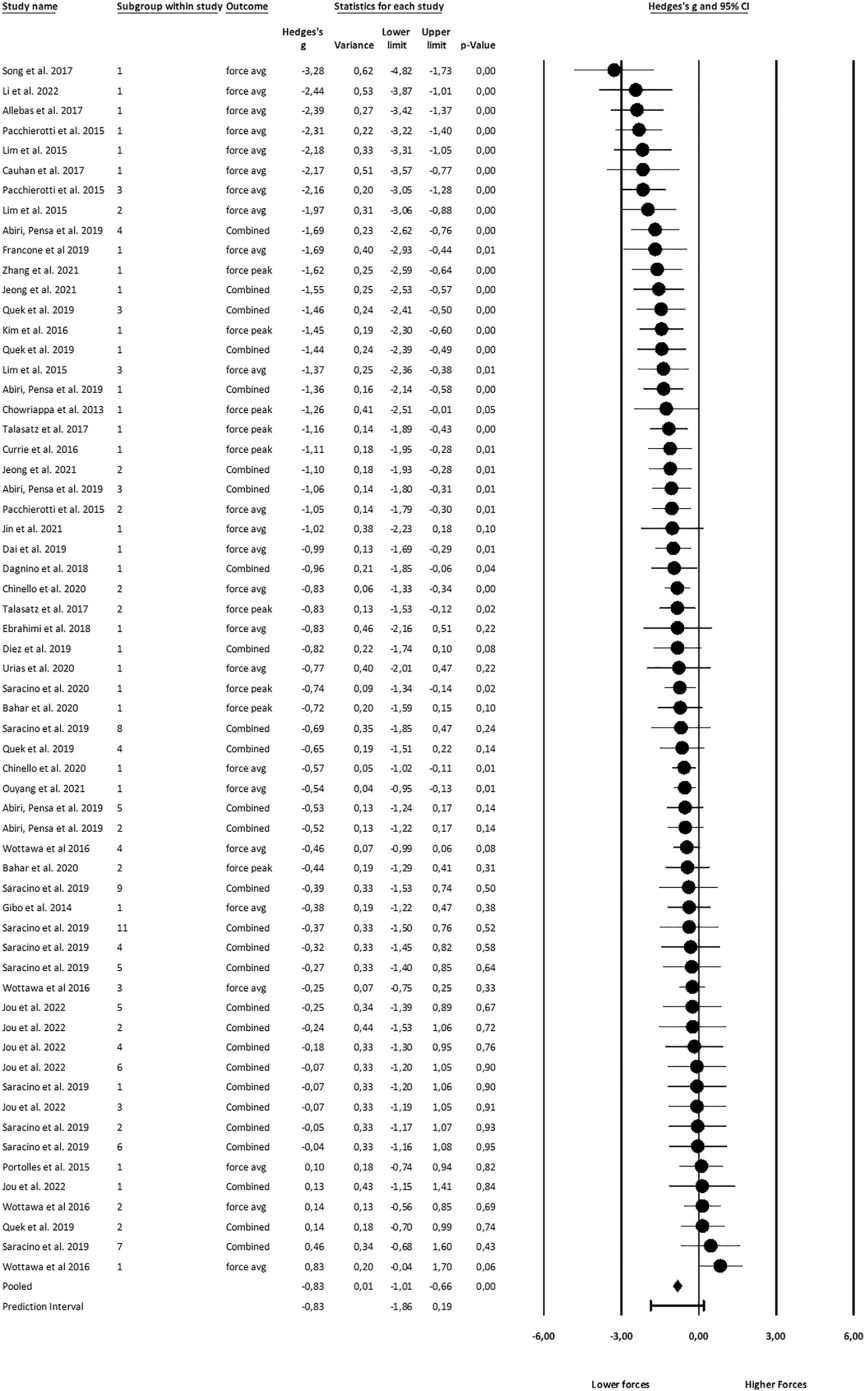
Effects on haptic feedback on applied force.

**Figure 3:**
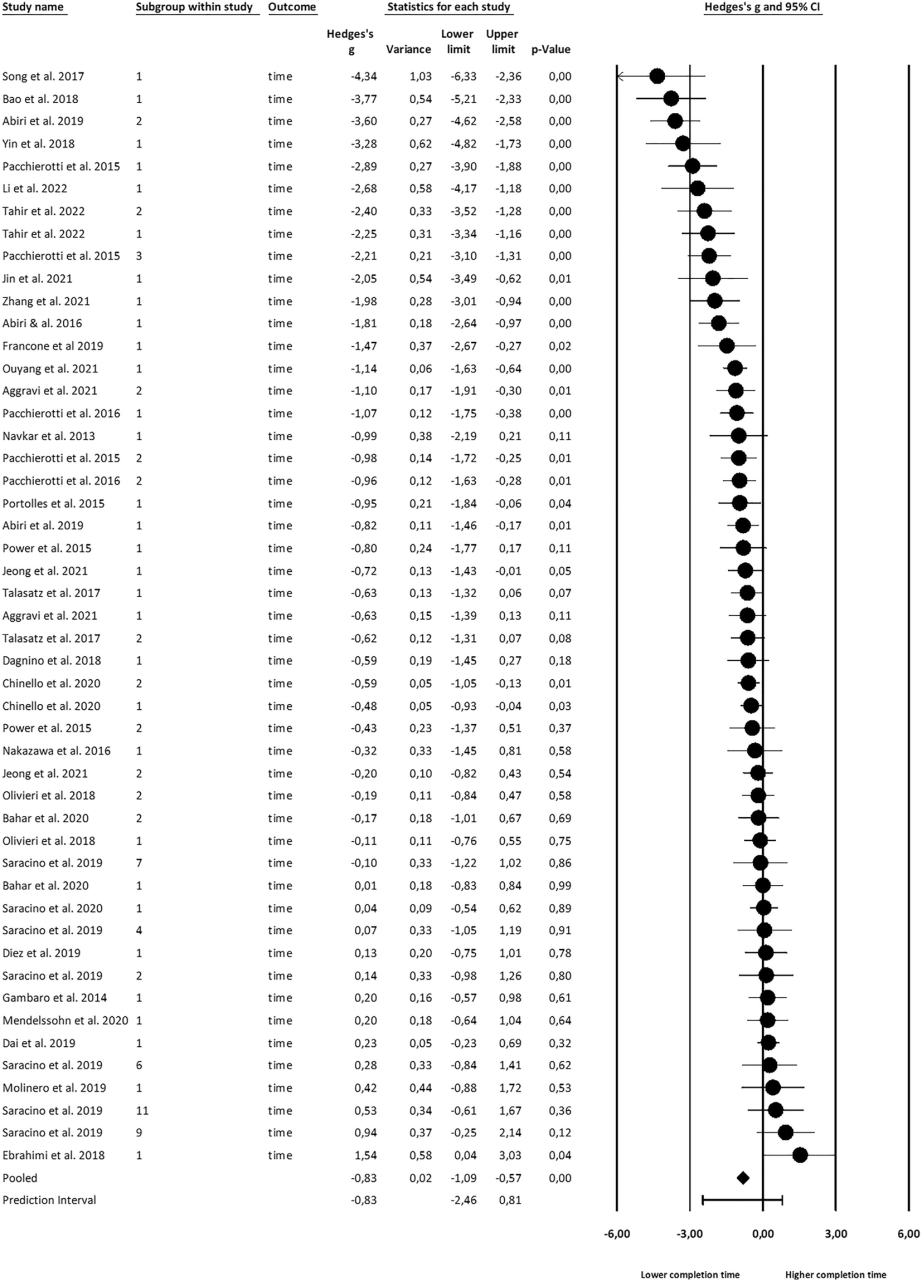
Effects on haptic feedback on completion time.

**Figure 4:**
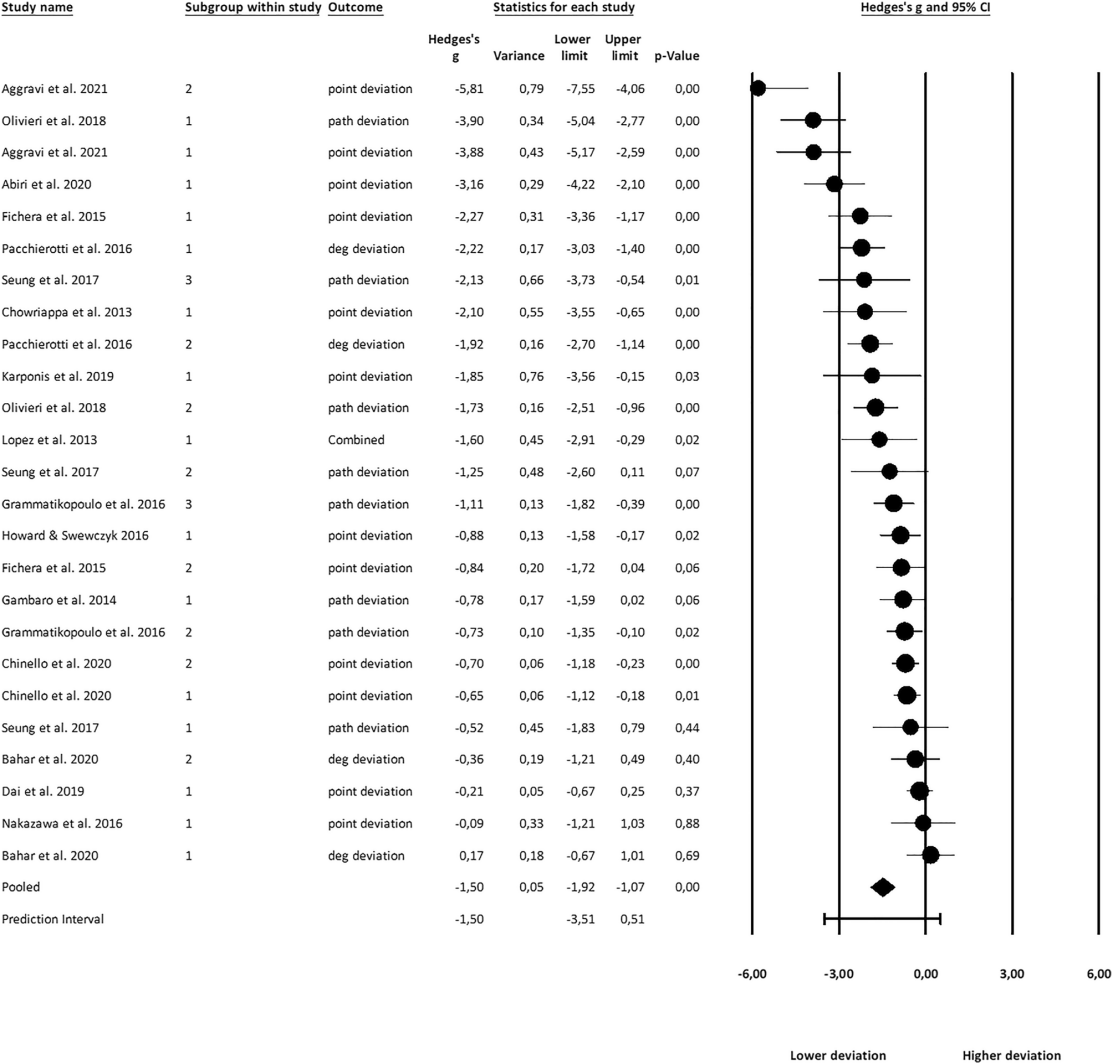
Effects of haptic feedback on accuracy.

Figures 2, 3 and 4 show the effect sizes, their variances, and overall prediction and confidence intervals. Forest plots have been created in CMA. Note that effect sizes in the plots are natively rather than valence-coded due to software limitations.

### Effects on applied force

#### Main effects

##### Average forces

Based on the analysis of k = 52 study groups comprised of a total of n = 249 individuals, a strong effect of haptic feedback on the average forces was found (Hedges’ g = 0.83, CI 0.63–1.03). The Q-test indicates that effect sizes varied substantially between studies (Q(51) = 135.95, p < 0.001). 62% of that difference in effect size is accounted for by the heterogeneity of the true effect rather than random error (*I*^2^ = 0.62).

##### Peak force

K = 37 study groups with a total of n = 172 individual subjects were analyzed. A moderate effect on peak forces was found (g = 0.69, CI 0.51–0.87). True effect sizes differed in the population (Q(36) = 51.7, p = 0.043). *I*^2^ = 30% of the observed difference can be attributed to the difference in true effect size.

##### Combined force

The effect size for combined average and maximum applied forces was also calculated. For study groups that reported both outcomes, combined means were used in line with the recommendations of Borenstein et al.^31^. k = 61 Study groups were analyzed. The overall effect on force was large (g = 0.83, CI 0.66–1.01). True effect sizes in the population varied (Q(60) = 147.20, p < 0.001), and this variance accounts for *I*^*2*^ = 59% of the observed variance.

#### Moderation

Only combined force was considered for moderation effects to achieve a reasonable number of observations per cell. Only 46 study groups could be analyzed for expertise level due to lacking data reports in the primary studies. Significant differences (Q(2) = 13.05, p < 0.001) between levels of subjects’ expertise were found. Significant effects were found for inexperienced (g = 0.98) and novice subjects (g = 0.84) but not for experts.

Force data was reported for seven different tasks, and significant effect size differences (Q(6) = 16.9, p < 0.001) emerged, with effect sizes being the largest for catheterization tasks (g = 1.72). Only for section tasks, no significant effect of haptic feedback was found.

No study using vibrotactile feedback reported data for applied forces. Effect sizes were different between subgroups (Q(2) = 17.92, p < 0.001), being largest for combined (g = 1.39) and smallest for cutaneous feedback (g = 0.36). As Virtual Fixtures aim to provide haptic feedback before contact occurs, no comparison between Virtual Fixtures and Contact Forces can be made. For a full list of effects, refer to Table 2.

**Table 2:** Effect size of haptic feedback on applied forces overall and across subgroups.

Group	Observations	Hedges’ g	95% CI	p-value	Q-value
Overall	61	0.83	**0.66 to 1.01**	** < 0.01**	
Expertise					
Experienced	10	0.15	− 0.18 ** to** 0.47	0.37	
Novice	13	0.68	**0.44 to 0.93**	** < 0.01**	
Inexperienced	23	0.98	**0.65 to 1.31**	** < 0.01**	
Total between	3			** < 0.01**	13.05
Task					
Catheter	5	1.72	**0.95 to 2.50**	** < 0.01**	
Grasp	29	0.88	**0.59 to 1.17**	** < 0.01**	
Insertion	4	0.87	**0.43 to 1.31**	** < 0.01**	
Palpation	7	0.70	**0.35 to 1.04**	** < 0.01**	
Section	9	0.16	− 0.23 ** to** 0.55	0.42	
Suturing	3	0.96	**0.54 to 1.39**	** < 0.01**	
Tracing	4	0.83	**0.08 to 1.57**	**0.03**	
Total between	7			**0.01**	16.92
FB Type					
Cutaneous	18	0.36	**0.11 to 0.61**	**0.01**	
Kinesthetic	37	0.96	**0.75 to 1.18**	** < 0.01**	
Combined	6	1.39	**0.83 to 1.96**	** < 0.01**	
Total between	3			< **0.01**	17.92

Significant values are in [bold].

Columns from left to right denote the subgroup, the number of observations per subgroup, the effect of providing haptic feedback, the confidence interval of the effect size, and its p-value. The total between the p-value and Q-value indicates whether the effect sizes differ significantly between subgroups.

### Effects on completion time

#### Main effects

An analysis of n = 372 subjects across k = 49 study groups found a large effect (g = 0.83, CI 0.57–1.09). As for forces, the effects on time showed a significant heterogeneity (Q(48) = 229.10, p < 0.001), accounting for *I*^*2*^ = 79% of the observed variance.

#### Moderation

36 observations reported subjects’ expertise. Significant effect size differences (Q(2) = 37.46, p < 0.001) between levels of expertise were found, with time reduction only manifesting for inexperienced subjects (g = 1.21).

For the eight different tasks, effect sizes differed significantly (Q(7) = 10.98, p < 0.001). Significant effects were found for catheterization (g = 2.07), grasping (g = 1.30), insertion (g = 0.53) and palpation (g = 0.69).

No significant effect size difference between feedback types was found. Similarly, contact force rendering and virtual fixtures provided significant time savings but did not differ significantly. Full results are reported in Table 3.

**Table 3:** Effect size of haptic feedback on completion time across subgroups.

Group	Observations	Hedges’ g	95% CI	p-value	Q-value
Overall	49	0.83	**0.57 to 1.09**	** < 0.01**	
Expertise					
Experienced	7	0.38	− 0.37 to 1.12	0.32	
Novice	5	-0.24	− 0.55 to 0.06	0.12	
Inexperienced	24	1.21	**0.86 ** to** 1.56**	** < 0.01**	
Total between	3			** < 0.01**	37.46
Task					
Catheter	11	2.07	**1.30 to 2.84**	** < 0.01**	
Grasp	8	1.30	**0.62 to 1.97**	** < 0.01**	
Insertion	5	0.53	**0.16 to 0.90**	** < 0.01**	
Laser	2	0.15	− 0.32 to 0.61	0.53	
Palpation	11	0.69	**0.21 to 1.17**	** < 0.01**	
Section	4	-0.18	− 0.69 to 0.34	0.50	
Suturing	3	0.15	− 0.68 to 0.98	0.72	
Tracing	4	0.22	− 0.31 to 0.74	0.42	
Total between	8			** < 0.01**	10.98
FB type					
Cutaneous	6	0.97	**0.63 to 1.30**	** < 0.01**	
Kinesthetic	35	0.67	**0.35 to 0.98**	** < 0.01**	
Vibrotactile	6	1.26	**0.18 to 2.34**	**0.02**	
Combined	2	1.69	− 0.56 to 3.94	0.14	
Total between	4			0.42	2.81
VF vs. CF					
Contact Force	38	0.90	**0.59 to 1.20**	** < 0.01**	
Virtual Fixture	11	0.59	**0.11 to 1.07**	** < 0.01**	
Total between	2			0.30	1.09

Significant values are in [bold].

### Effects on accuracy

#### Main effects

K = 25 study groups with n = 192 individuals were analyzed. Of these 25 observations, 13 had natively reported point-deviation, 7 path-deviation, 4 degree-deviation, and 1 had already natively aggregated these measures (see Fig. 4). An overall effect of g = 1.50 (CI 1.07–1.92) was found. The true effect sizes varied in the population (Q(24) = 145.94, p < 0.001). The variance of the true effect accounted for *I*^*2*^ = 0.83 of the observed variance.

For 20 study groups, expertise was reported, but no effect size difference between levels of expertise was found. Interestingly, the observed effect was highest for experts (g = 2.78), but the sample was very small, with only k = 2 expert groups that reported accuracy measures.

Accuracy measures for seven tasks were reported. A significant effect for haptic feedback was found for every task except for suturing. However, the size of the effects did not differ significantly from each other.

Comparing feedback types, significant effects were found for every type except combined feedback. Again, the number of groups in this category was rather small (k = 2). No significant effect size difference between groups was found.

A comparison of the effect sizes between Virtual Fixtures and Contact Forces showed significant effects of haptic feedback for both groups. There was no significant difference in effect sizes. A full list of results can be found in Table 4.

**Table 4:** Effect size of haptic feedback on accuracy across subgroups.

Group	Observations	Hedge’s g	95% CI	p-value	Q-value
Overall	**25**	**1.50**	**1.07 to 1.92**	** < 0.01**	
Expertise					
Experienced	2	2.78	**0.65 to 4.90**	**0.01**	
Novice	9	1.20	**0.61 to 1.78**	** < 0.01**	
Inexperienced	9	1.87	**1.07 to 2.67**	** < 0.01**	
Total between	3			0.20	3.23
Task					
Insertion	7	2.12	**0.68 to 3.57**	** < 0.01**	
Laser	4	2.14	**0.97 to 3.31**	** < 0.01**	
Palpation	4	1.30	**0.57 to 2.03**	** < 0.01**	
Scan	1	1.11	**0.39 to 1.82**	** < 0.01**	
Section	2	1.62	**0.59 to 2.65**	** < 0.01**	
Suturing	3	1.34	− 0.11 to 2.80	0.07	
Tracing	4	0.63	**0.20 to 1.05**	** < 0.01**	
Total between	7			0.09	10.98

FB type	Observations	Hedges’ g	95% CI	p-value	Q-value
Cutaneous	2	1.25	0.00 to 2.49	0.05	
Kinesthetic	16	1.30	**0.75 to 1.84**	** < 0.01**	
Vibrotactile	5	2.40	**1.08 to 3.73**	** < 0.01**	
Combined	2	1.40	− 0.12 to 2.93	0.07	
Total between	4			0.50	2.39
VF vs. CF					
Contact Force	12	1.76	**1.06 to 2.45**	** < 0.01**	
Virtual fixture	13	1.28	**0.78 to 1.78**	** < 0.01**	
Total between	2			0.28	1.19

Significant values are in [bold].

#### Success rates

Lastly, success rates showed, based on the analysis of k = 15 study groups with n = 181 subjects, a large effect of g = 0.80 (CI 0.24–1.35). Substantial heterogeneity was present (Q(14) = 71.33, p < 0.001), which accounted for *I*^*2*^ = 8037% of the observed variance. The prediction i3dnterval is PI − 1.36 to 2.96. Due to the small number of observations for this measure, most of which fall under the same paradigm (i.e., palpation), this measure had to be excluded from subgroup analysis.

#### Publication bias

To assess a potential publication bias, the classic Fail-Safe-N was calculated for force, time, and accuracy measures. It indicates how many studies with an effect size of zero would have to be found for the meta-analysis to no longer be significant on a level of p < 0.05^80^. The test returned a value of N = 9627. This means that 9627 hypothetical unpublished or otherwise missed papers with no effect for haptic feedback had to exist to invalidate the findings of this analysis via publication bias. Although it is important to acknowledge the limitations of this measure, given the large number, potential bias can be deemed too weak to fundamentally alter results. A visual inspection of the funnel plot (see Appendix 1) indicates no strong bias either. If bias is present in the sample, the analysis can be expected to react robustly.

## Discussion

To quantify the effect of haptic feedback on surgical performance, the results of 56 primary studies from 2013 onwards were aggregated using meta-analytical methods. Significant overall effects were found for all investigated measures: applied forces, completion time, accuracy, and success rates. However, the present study goes beyond a simple analysis of the main effect. To identify the specific conditions under which the benefits of haptic feedback are largest, the moderating effects of expertise, task, and feedback type were investigated via subgroup analyses. The subject’s experience and the specific nature of the task were identified to have the greatest influence on how beneficial haptic feedback is.

Large overall effects were found across paradigms and measures. The effects found in the present study were larger than those described by Weber and Eichberger^25^ in an earlier meta-analysis. In particular, the present study's effects on completion time (g = 0.83 vs. g = 0.22) and accuracy (g = 1.5 vs. g = 0.69) were substantially larger. This is likely to reflect both technological progress and an application of haptic feedback to new surgical procedures (e.g., catheterization tasks). This underscores haptic feedback's continued and expanding role in medical practice.

A strong effect on applied forces was found overall and in most subgroups, with a notable exception being experts. This finding is of critical importance as some research suggests higher rates of patient tissue damage during RAS^6^, which has been linked to higher interaction forces^79^. This may be the most considerable benefit of haptic feedback for robot-assisted procedures. Furthermore, the effects were largest when combined feedback was employed, suggesting it is a fruitful avenue of development.

Effects on completion times were mixed, with no significant effects of haptic feedback for tracing, suturing, and laser surgery tasks. This is also in line with inconsistent findings of previous meta-analyses: Nitsch and Färber^80^, e.g., found a significant effect for completion time (g = 0.75), whereas Weber and Schneider^81^ did not report such an effect. This might be because the added guidance gives users the security necessary for some tasks to complete the task quickly. However, for other tasks, this might be canceled out by the haptic feedback, prompting a more cautious behavior. This is corroborated by the higher accuracy and reduced force application found in the present study.

Effects on Accuracy were overall largest and crucially the only metric on which significant effects for experts were found. Due to the very small number of studies in this subgroup (k = 2), this finding should be interpreted cautiously.

Overall, the level of expertise emerged as an important moderator, with effects generally smaller for experts. This is consistent with expectations among practitioners and researchers suggesting that long-term practitioners of RAS develop techniques to partially compensate for the loss of haptic via their practice^82^. However, whereas, e.g., Hagen et al.^21^ suggest that even beginning surgeons can compensate for missing haptic feedback by visual cues alone, this meta-analysis found substantial effects for novices. Combined with results suggesting that haptic feedback enhances surgeons' training^6^, it can be constituted that especially less experienced surgeons benefit from haptic feedback systems.

This study found that haptic feedback benefits depend on the task demands, with the strongest effects for catheterization tasks and the weakest effects for sectioning tasks. Catheterization is a relatively new field of applications for haptic feedback. During vascular catheterization, the surgeon has to rely on low-fidelity vision provided by MRT or X-ray scans while performing a task that requires extreme caution to prevent injuring the patient. Based on the strong positive effects on completion time and force application found in this review, it can be predicted that haptic feedback will play a crucial role in this intervention. Especially considering the use of x-ray imaging is a strong motivator for reduced patient exposure times and the fact that instrument contact forces well below one Newton can lead to vascular puncturing^83^.

Benefits on all measures were found for grasping and palpation tasks. This is an important finding considering the high prevalence of laparoscopic interventions among RAS, where these tasks play a crucial role^22^. For suturing—another common laparoscopic task—only force regulation strongly benefitted from haptic feedback. This is, however, crucial to prevent the common problem of suture breakage. Many researchers have argued that the widespread adoption of haptic feedback systems would greatly benefit this robot assisted laparoscopy^4,20^. The present study substantiates this argument.

Insertion tasks showed substantially reduced forces and completion time and improved accuracy when haptic feedback was available. In non-robot-assisted surgery, the shear forces incurred by different layers of tissue are the primary means for surgeons to guide the correct insertion of instruments^10^. Artificial haptic feedback restores this method for RAS.

Tracing, a task common in retinal surgery, showed strong effects for force and accuracy but not for time. Given that in retina surgery, time is usually a less critical metric due to the absence of time-constraining circumstances like heavy blood loss, these results are nonetheless encouraging.

For the other tasks (sectioning, laser surgery, and scanning), results were comparatively mixed but still overall positive (except for sectioning tasks). For these tasks, advancements in stereoscopic 3D vision and visual feedback may already provide enough guidance for surgeons, as suggested by the findings of Weber and Schneider^80^.

That no form of haptic feedback emerged clearly superior contrasts with the findings of Weber and Eichberger^25^, who found larger effect sizes for kinesthetic feedback than for vibrotactile feedback. The studies of the present meta-analysis skewed heavily away from the latter modality, with only k = 11 observations for pure vibrotactile feedback. The analysis by Weber and Eichberger^25^ included a total of k = 55 observations for this feedback type. This discrepancy in data may explain the different findings. Furthermore, this indicates a trend away from pure vibrotactile feedback in favor of kinesthetic and combined feedback. Future studies could integrate the data of several meta-analyses on the topic to further investigate the differences.

No significant effect size difference was found between haptic feedback and virtual fixtures. There is the potential of an interaction here precluding an effect, as a look into the studies revealed that virtual fixtures were more commonly combined with vibrotactile feedback and force rendering more commonly with kinesthetic feedback (see Table 1).

In fact, a deeper three-way interaction between measure, task, and feedback can be conceived. For example, vibrotactile feedback might be more effective in reducing completion time than kinesthetic feedback, specifically for palpation tasks^31^. The present study, however, lacks the power to test for such interactions.

Another potential limitation of this analysis is that several related sub-measures had to be aggregated as each sub-measure by itself had too few observations per factor level to allow for subgroup analysis. Combining similar outcomes to organize and investigate large data sets is a core purpose of meta-analyses, but in doing so, the comparability of data must be considered^31^. Since peak and average forces just constitute two different data points of the same measurement, comparability can be readily assumed. In the case of deviation, it can be noted that the different sub-measures are inherently linked. During a needle insertion task, the surgeon may have to guide the instrument’s tip to an exact location to administer a substance precisely and avoid unnecessary tissue damage^36^. In this example, deviation from this target point and deviation from the ideal angle of insertion are intrinsically linked, and combining them can, therefore, be considered valid.

Lastly, there is the potential that the image-based retrieval mentioned in the method section, made necessary by lacking direct reporting in the primary studies, is less accurate than direct numerical retrieval. A total of 33 observations in the final study were retrieved this way. To test whether this retrieval method has skewed the data, the effect size of all image-retrieved outcomes was compared to the effect size of all numerically retrieved outcomes. If the image-based retrieval leads to comparable reliability as direct numerical retrieval, no difference in effect sizes should exist. Indeed, no significant difference in effect sizes was found (Q(1) = 0.11, p = 0.74). It was thus concluded that the retrieval method yielded sufficient reliability, and image-retrieved data was included in the analysis.

## Conclusion

With the benefits of haptic feedback firmly established and application fields where its impact is especially strongly identified, research can focus on these fields to investigate further factor interactions and compare different forms of haptic feedback. In particular, a comparison between direct haptic feedback and haptic substitution to identify in which scenarios one is preferable over the other appears to be a fruitful next step (Table 5).

**Table 5:** Summary of most important results.

Key takeaways	
Main effects	Large positive effects of providing haptic feedback were found for all measures: applied force (g = 0.83), completion time (g = 0.83), accuracy (g = 1.50), and success rates (g = 0.80)
Level of expertise	Expertise moderates the effects found for applied forces and completion time but not for accuracy. Generally, the difference between haptic and no haptic feedback is larger for less experienced users
Task	The type of task likewise moderates the effects found for forces and completion time but not for accuracy. In both cases, the difference between haptic and no haptic feedback was largest for catheterization and smallest for cutting tasks
Feedback type	How the haptic feedback was delivered only affected the effects found for forces. Cutaneous feedback had the weakest (g = 0.36) and combined feedback the strongest effect (g = 1.39)
Virtual fixtures vs. contact forces	Whether the feedback was given about actual physical contact or virtual fixtures did not affect effect size

In summary, providing haptic feedback was found to have desirable effects across task and feedback paradigms. The effects are diminished for practitioners with high levels of expertise but remain descriptively present. Reduced interaction forces and completion times have great potential to limit tissue damage and blood loss during the operation and thereby vastly improve patient safety. These findings can serve as a basis to inform equipment acquisition and future research directions.

### Supplementary Information

Supplementary Information.

## Data Availability

All data generated during this study is published in this article. All primary studies analyzed in this meta-analysis have been reported and are publicly available.
