# Supplementary material for: The benefits of haptic feedback in robot assisted surgery and their moderators: a meta-analysis
Source: Sci Rep. 2023 Nov 6;13:19215. doi: 10.1038/s41598-023-46641-8 (PMC10628231; doi:10.1038/s41598-023-46641-8)
Supplement: Supplementary file 1 — Supplementary Information. [file 41598_2023_46641_MOESM1_ESM.docx]

**Appendix**


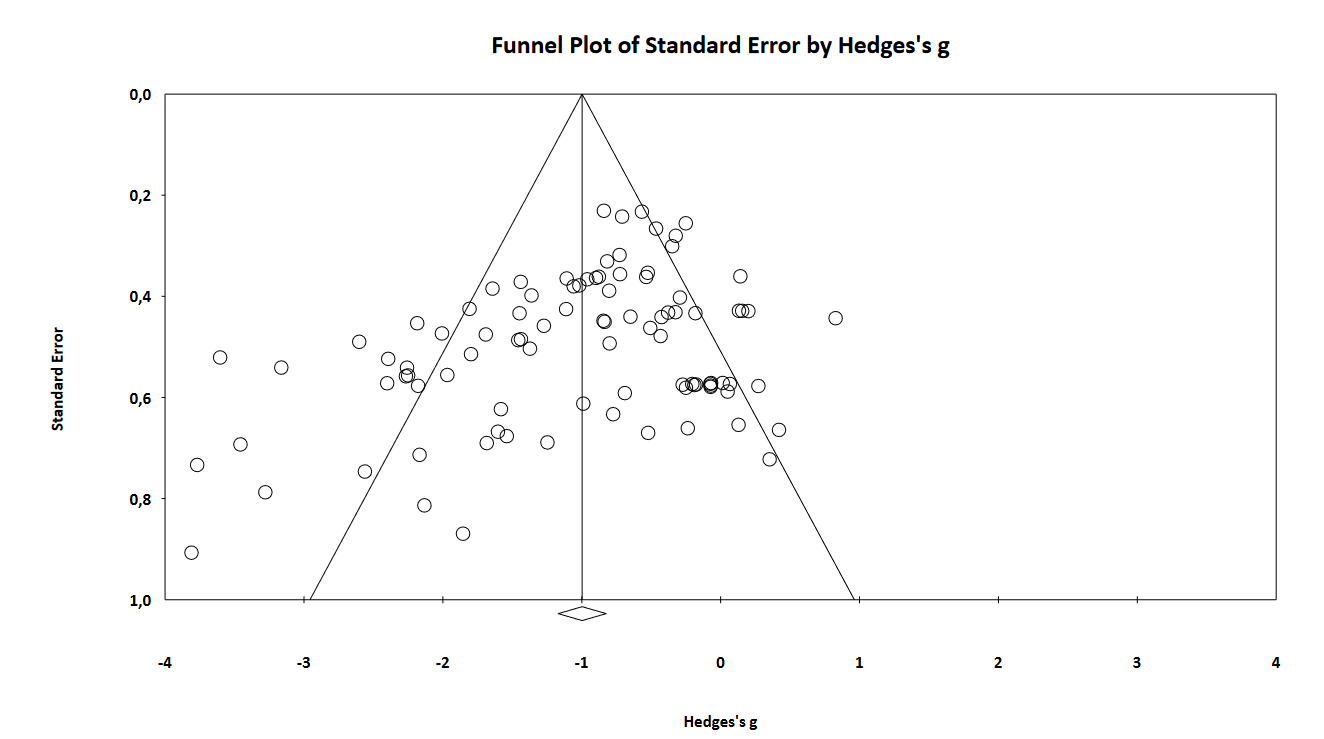


**Appendix 1:** Funnel plot of effect sizes for force, time and accuracy. Figure was created with CMA.
